# The Association between Blood SIRT1 and Ghrelin, Leptin, and Antibody Anti-Hypothalamus: A Comparison in Normal Weight and Anorexia Nervosa

**DOI:** 10.3390/jpm13060928

**Published:** 2023-05-31

**Authors:** Andrea Amerio, Andrea Escelsior, Eleonora Martino, Antonella Strangio, Andrea Aguglia, Matteo Marcatili, Benedetta Conio, Samir Giuseppe Sukkar, Daniele Saverino

**Affiliations:** 1 Department of Neuroscience, Rehabilitation, Ophthalmology, Genetics, Maternal and Child Health (DiNOGMI), Section of Psychiatry, University of Genoa, 16132 Genoa, Italy; andrea.amerio@unige.it (A.A.); andrea.escelsior@unige.it (A.E.); andrea.aguglia@unige.it (A.A.); 2 IRCCS Ospedale Policlinico San Martino, 16132 Genoa, Italy; eleonora.martino@unige.it (E.M.); benedetta.conio@hsanmartino.it (B.C.); samir.sukkar@hsanmartino.it (S.G.S.); 3 Dietetics and Clinical Nutrition Unit, University of Genoa, 16132 Genoa, Italy; 4 Department of Experimental Medicine (DiMeS), Section of Human Anatomy, University of Genoa, 16132 Genoa, Italy; antonella.strangio@unige.it; 5 Department of Mental Health, Fondazione IRCCS San Gerardo dei Tintori, 20900 Monza, Italy; matteo.marcatili@gmail.com

**Keywords:** serum SIRT1, leptin, ghrelin, anti-hypothalamic antibody, anorexia nervosa

## Abstract

Sirtuin 1 (SIRT1) is a sensor of cell energy availability, regulating metabolic homeostasis as well as leptin and ghrelin, and it could be considered as a potential plasmatic marker. The aim of this study was to assess whether circulating SIRT1 varies consistently with leptin, ghrelin, body mass index (BMI), and IgG reactive to hypothalamic antigens in anorexia nervosa (AN). Fifty-four subjects were evaluated: 32 with AN and 22 normal-weight control subjects. Serum levels of SIRT1, leptin, ghrelin, and IgG reactive to hypothalamic antigens were evaluated by ELISA. Results showed that serum SIRT1 is increased in patients with AN, and the amount is decreased in relation to the duration of the illness. SIRT1 concentration approaches the values obtained for the control group, although the difference is still statistically significant. A negative correlation between serum SIRT1 values and leptin or BMI values has been found. On the contrary, a positive correlation between SIRT1 and ghrelin or IgG specific for hypothalamic antigens is reported. These findings suggest that a peripheral evaluation of SIRT1 could be a possible clinical/biochemical parameter related to AN. In addition, we can assume that SIRT1 is related to autoantibody production and may correlate with the intensity/severity of AN. Thus, reducing the production of autoantibodies specific for hypothalamic cells could be a sign of improvement of the clinical condition.

## 1. Introduction

Anorexia nervosa (AN) is a severe and chronic psychiatric disorder that mainly involves adolescents and adults. Its etiopathogenesis is still not entirely clear. Diagnosis is defined according to the Diagnostic and Statistical Manual of Mental Disorders-fifth edition (DSM-5), and the clinical picture consists of reduced caloric and food intake, fear of weight gain, and a disturbed image of one’s own body [1]. The interaction among biological, genetic [2,3,4], cognitive [5,6] psychosocial, and social variables [7] was studied as etiological factors. The lifetime prevalence of AN is about one out of 200 females. Approximately 95% of individuals with an eating disorder are very young, usually from 12 to 25 years old. Although the F:M ratio of eating disorders is about 9:1, the incidence in males is increasing. Furthermore, approximately one half of patients with AN fully recover, about one third achieve only partial recovery, and a fifth remain chronically ill. AN has the highest mortality rate of any mental health disorder, with an estimated all-cause standardized mortality ratio of 1.7 to 5.9 [7].

Recent studies also imply altered immune response in patients with AN [8,9,10,11]. The correlation between the immune system and nutrition has been analysed from two different points of view. Initially, studies investigated the effect of restricted food intake on the immune response. Subsequently, it has been hypothesized that clinical conditions of primary or secondary dysregulation of the immune state (i.e., infections or autoimmune diseases) could lead to AN. Malnutrition causes a decrease in some factors considered fundamental for an adequate immune response [12]. Phagocytic function, complement system, cytokine production, mucous secretory antibody response, antibody affinity, and mediated cell immunity have been identified as potential significant alterations [12]. Therefore, it is intuitive that dysregulation of the immune system and the increased risk of infection are potentially associated with chronic AN [13]. Several blood alterations, including leukopenia, polycythemia, and thrombocytopenia, have also been found in young male patients with AN [14]. Furthermore, elevated proinflammatory cytokines, with increased levels of tumor necrosis factor (TNF)-α, interleukin (IL)-1α, and IL-6 [15,16], phenotypes associated with modulation of the cellular components of the adaptive and innate immune system, have been reported in patients with AN [11]. Therefore, the significant role of cytokines in the modulation of inflammation, control of infections, regulation of neurotransmitter systems, neuroplasticity, and neuroendocrine functioning is recognized [11]. Thus, the observation that inflammation varies with the duration of the disease seems to validate the hypothesis that the role of the immune system can actually be causal in the pathogenesis and maintenance of AN [16]. It has been suggested that autoimmunity may be one of the pathogenetic causes of AN. Several studies highlight the production of autoantibodies against regulatory peptides and hypothalamic neurons [10,11,17,18,19]. These autoantibodies would induce an appetite disorder with inadequate nutritional and caloric intake. In fact, the presence of autoantibodies to hypothalamic cells in the serum of anorexic subjects and their positive correlation with ghrelin, pro-opiomelanocortin (POMC), and agouti-related peptide (AGRP) levels, but negatively with leptin, was demonstrated [10]. On the other hand, a higher rate of autoimmune clinical conditions is demonstrated among patients with eating disorders: juvenile systemic lupus erythematosus [20], celiac and inflammatory bowel diseases [21,22,23], Hashimoto’s thyroiditis [24,25], Raynaud phenomenon, and scleroderma [26]. These findings reinforce the hypothesis of shared pathogenetic mechanisms that could also trigger each other and that the resolution of AN could be linked to a possible immunosuppressive treatment.

The hypothalamus, through the arcuate nucleus (ARC), receives signals from the periphery and processes homeostatic responses to peripheral tissues, and it is considered the “main hypothalamic center” for feeding control [27]. ARC hypothalamic neurons respond to peripheral nutritional components (i.e., glucose and fatty acids) and hormones (i.e., leptin and ghrelin), the latter regulating food intake and body weight [10]. Leptin, a fat-dependent hormone, works as an afferent signal in a negative hypothalamic feedback loop with the aim of maintaining control of body fat [3]. In the case of excess fat, leptin has an anorexic action that reduces the probability to gain too much weight. Energy restriction provokes a decrease in serum leptin, with a consequent pro-inflammatory state related to the disease [28]. In contrast, ghrelin is a peptide derived from the stomach that increases food intake. So, leptin and ghrelin have an important and opposite role on energy balance [27].

Silent coupling information type regulation 2 homologous 1 (SIRT1) is a nicotinamide adenine dinucleotide (NAD+)-dependent enzyme, responding to stress and available nutrients [29]. During prolonged fasting, hunger, or severe restricted food intake, NAD+ increases levels [30], leading to the activation of SIRT1 [30]. SIRT1 translates these nutrient-limiting signals into changes in gene expression, determining cellular responses and organisms [31]. For example, restricted food intake induces hyperactivity in rodents, while this is not detected in animals without SIRT1 [32,33]. Similarly, animals overexpressing SIRT1 are more active during stressful environmental conditions [34]. These observations are particularly interesting, because hyperactivity and prolonged and too much exercise are often present in patients with AN. In addition, SIRT1 appears to be involved in the development of dependence [35], anxiety [36,37], and depression [37,38,39], which are clinical dimensions present in AN. Thus, SIRT1 could play a key role in the pathology of AN. Finally, recent studies have shown an upregulation of the expression of SIRT1 in AN, both in animal models [40] and in humans [41]. In addition, it has been demonstrated that the brain-specific KO of SIRT1 in mice is protective against the anorexic phenotypes when subjected to the activity-based anorexia model, while the brain-specific overexpression leads to worsened phenotypes, having an opposite effect. For these reasons, SIRT1 inhibition could be considered a potential target for the treatment of patients with AN [42].

The prevalence of AN is rising as well as its complications [43]. Several therapeutic approaches have been discussed for weight loss and its negative consequences [44]. Although all lead to improvement in many cases, none are always effective. Alterations involving leptin and SIRT1 can lead to alteration of the energy balance. A better understanding of regulatory mechanisms could help in the development of novel intervention tools, so it is crucial to study this important topic.

In this study, plasma concentrations of SIRT1, ghrelin, and leptin in patients with AN, and in subjects with normal weight, were analyzed in order to evaluate the possible association among these parameters. Furthermore, the correlations between SIRT1 and the presence of autoantibodies specific for hypothalamic cells in patients affected by AN was evaluated.

## 2. Materials and Methods

### 2.1. Study Design and Population

Participants (32 patients with AN and twenty-two in the healthy control group) were recruited from October 2019 to December 2022 at the Dietetics and Clinical Nutrition Unit, University of Genoa, and IRCCS Ospedale Policlinico San Martino, Genoa, Italy, for the treatment of AN. A total of 22 patients had restrictive AN, while 8 had purging phenotype (vomiting or use of laxatives). In addition, 2 males were enrolled. The primary diagnosis of AN was according to DSM-5 diagnostic criteria [1]. The healthy control group, matched by gender and age with patients with AN, consisted of 18 healthy women and 4 men not affected by any eating disorders, autoimmune diseases, or chronic clinical conditions.

Blood exams were made between 7:30 am and 9:30 am, before breakfast, and stored frozen until use without any freezing and thawing.

The Ethical Committee of IRCCS Ospedale Policlinico San Martino approved the study (CER 82/13 Emend. 028), and participants signed their written informed consent.

### 2.2. Serum SIRT1, Ghrelin, and Leptin Assay

SIRT1 and leptin sera concentrations were measured after an overnight fast. SIRT1 levels were determined according to the manufacturer’s instructions using quantitative ELISAs (MyBioSource, Cod. GDMBS705558). The inter- and intra-assay coefficients of variation were <8% and <12%, respectively, and the lower sensitivity was 5 pg/mL.

Quantitative determination of human leptin was carried out by the ELISA kit (MyBioSource, Inc., San Diego, CA, USA, Cod. MBS9425103). The sensitivity was <60 pg/mL, and inter- and intra-assay coefficients of variation were <6.5% and <7.8%, respectively.

Finally, the ELISA kit was used to measure serum ghrelin, which had the lowest sensitivity threshold of 0.31 ng/mL, and the intra-assay and inter-assay CV were 3.2% and 3.5%, respectively. For samples with a serum ghrelin concentration higher than 20 ng/mL, the ELISA tests were repeated using a greater dilution factor (1:100).

### 2.3. Anti-Hypothalamus Autoantibody ELISA Protocols

Levels of autoantibodies (IgG) specific for hypothalamic antigens were measured in the serum of AN patients and healthy controls by a direct ELISA method built in our laboratory [10]. Briefly, 96-well Maxisorb flat-bottom plates (Nunc) were coated overnight at 4 °C with a bovine hypothalamic lysate (Science Cell Research, cat#0613) (10 µg/mL in 50 µL/well). Subsequently, plates were incubated for 2 h with phosphate buffered saline (PBS) −3% bovine serum albumin (BSA) to avoid non-specific interactions. After washes, 100 µL of 1:100 in PBS−3% BSA diluted serum samples were plated in triplicate and incubated overnight at 4 °C with agitation. Then, wells were incubated with 100 µL of anti-human IgG HRP-conjugate (Jackson ImmunoResearch, as 1:10,000 in PBS−3% BSA buffer) for 45 min at room temperature. After incubation, plates were washed, and 100 µL of tetramethylbenzidine (TMB) substrates were added to develop colour for 15 min. Stop solution (100 µL) was then added, and plates were read at 450 nm for 15–20 min.

As a part of the assay, a standard curve for human IgG was carried out (of note, the standard curve was not specific for hypothalamic antigens, but exclusively for IgG) [10]. In addition, to gauge the overall reliability, the intra-assay % coefficient of variation and inter-assay % were calculated (respectively, 5% and 9.3%). Finally, deviation between triplicates was <10% for any reported value.

The lower sensitivity level was 30 ng/mL. Of interest, all of sera from AN were higher, whereas 8/22 control were lower the sensitivity level.

### 2.4. Statistical Analysis

The D’Agostino–Pearson normality test was used to test the normal distribution of our sample [45], quantifying how far the distribution is from Gaussian in terms of asymmetry and shape; then, it calculates how much each of these values differs from the value expected with a Gaussian distribution and computes a single p value from the sum of these discrepancies.

The Mann–Whitney U-test was performed to compare the levels of SIRT1. The Wilcoxon test was used to analyse differences in SIRT1 concentrations based on the duration of the psychiatric disorder (cut-off three years). Spearman regression analysis was used to investigate the relationship between SIRT1 levels and ghrelin, leptin, autoantibodies specific for hypothalamic antigens, or BMI. The statistical *p* value was set at <0.05. All statistical analyses were performed using GraphPad Prism 6.0 (GraphPad Software Inc., San Diego, CA, USA) software.

## 3. Results

### 3.1. Characteristics of the Study Population

The total sample included 2 males and 30 females, aged 18–62 years old, suffering from AN, and 4 males and 18 females, aged 18–51 years old, as the healthy control group. Table 1 shows the characteristics stratified according to their body mass index (BMI). Briefly, the mean BMI was 15.55 ± 1.98 kg/m^2^, and 20.57 ± 4.00 kg/m^2^ in the AN and control groups, respectively (*p* < 0.001).

Despite the small number of males enrolled in the study, no statistical differences with females were found (data not shown).

### 3.2. Serum SIRT1 Levels Are Increased in Patients with AN

**Figure 1:**
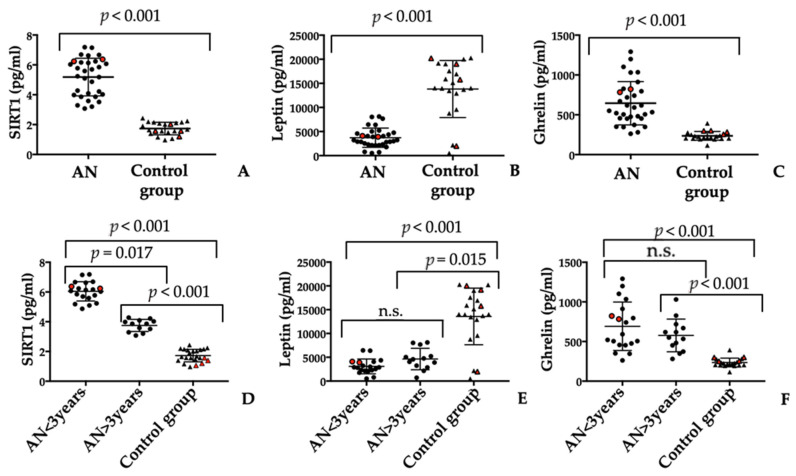
SIRT1 (**A**) and ghrelin (**B**) amounts are significantly increased in patients affected by AN compared to control group (CG). On the contrary, leptin (**C**) amount is significantly decreased in patients with AN compared to control group. In addition, the amount of serum SIRT1 (**D**), leptin (**E**), and ghrelin (**F**) correlated to the duration of illness. The statistical *p* value was set at <0.05 (n.s.: not significant). Red symbols represent males.

SIRT1 levels were statistically different between the AN and control groups (*p* < 0.001). Table 1 and Figure 1A show the data.

Looking at the graphical distribution of SIRT1 levels in the serum of patients with AN (Figure 1A), the possibility of dividing this population into two different groups is evident. By analysing the characteristics of the patients within the two clusters, the only difference is the duration that the psychiatric disorder was observed: less or more than 3 years. Therefore, the duration of AN could possibly affect serum levels of SIRT1. In fact, levels of SIRT1 decreased over time, approaching values in the healthy control group (Figure 1D). In detail, the amount of SIRT1 AN < 3 was 6.04 ± 0.65 pg/mL, whereas it was 3.75 ± 0.41 pg/mL for AN > 3 years (*p* = 0.019). As a baseline, SIRT1 in the control group was 1.72 ± 0.42 pg/mL. However, as can be depicted in Figure 1D, the differences between the amount of SIRT1 in AN>3 years and the control group are still statistically significative (*p* = 0.007).

Despite the small number of males recruited, there were no evident differences with females (as can be depicted in Figure 1A–F, red symbols represent males).

### 3.3. Serum Leptin and Ghrelin Molecules Evaluation

Serum leptin levels were statistically different among the study groups (*p* < 0.001). Mean serum levels of leptin were significantly lower in patients with AN than the control group (3715 ± 1994 pg/mL vs. 13,831 ± 5917 pg/mL; range 500–8110.0 pg/mL vs. 565.0–20,282.0 pg/mL, respectively). Table 1 and Figure 1B summarise the results.

Serum levels of ghrelin in the healthy control group and patients with AN are provided in Table 1 and Figure 1C. The mean serum level of ghrelin was significantly higher in patients with AN than in the healthy control group (645.3 ± 271.9 pg/mL vs. 234.5 ± 54.04 pg/mL; range 262.4–1292.0 pg/mL vs. 115.4–392.6 pg/mL).

Unlike SIRT1 serum values, the analysis of variations for both parameters showed no difference in relation to the duration of the psychiatric disorder (Figure 1E,F).

### 3.4. Anti-Hypothalamic Autoantibodies Can Be Detected in Serum from Patients with AN

The presence of autoantibodies reacting to hypothalamic antigens was evaluated by homemade ELISA assays. Concentrations of all anti-hypothalamus IgG antibodies were significantly higher in patients with AN than in the healthy control group (*p* < 0.001) (Table 1) while levels of autoantibodies were significantly lower in the healthy control group than in patients with AN (mean 167.5 ± 368.4 ng/mL vs. 8476.0 ± 2267.0 ng/mL; range 30.0–1350 ng/mL vs. 2883.0–11,988.0 ng/mL; *p* < 0.001).

Lastly, no significant association among age at onset or duration of the illness and anti-hypothalamus reactivity was found (data not shown). In addition, no gender statistical differences were evident (data not shown).

### 3.5. SIRT1 Relationship with Leptin, Ghrelin, BMI, and Antibody Anti-Hypothalamus

**Figure 2:**
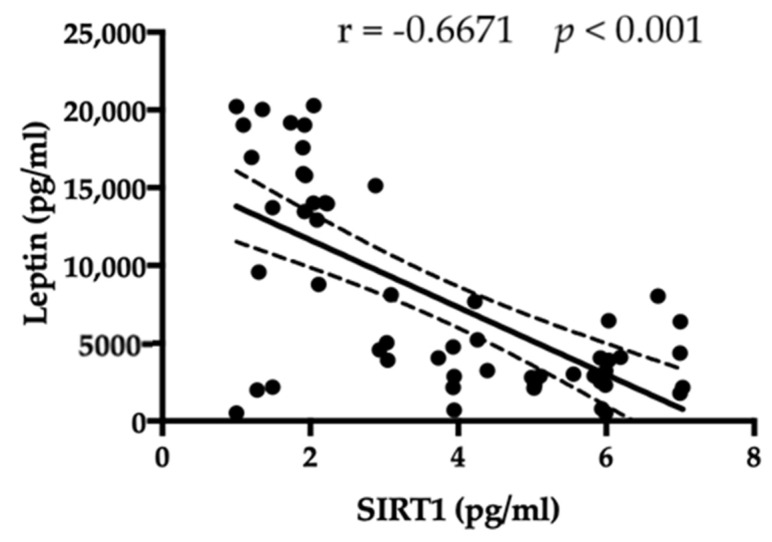
An inverse correlation among amount of SIRT1 and leptin is evident.

The correlation analysis among different analyses was performed. An inverse relationship between SIRT1 and leptin was observed (Figure 2). Thus, patients affected by AN reported the highest plasma levels of SIRT1 and the lowest concentration of leptin (Spearman r = −0.6671, *p* < 0.001).

**Figure 3:**
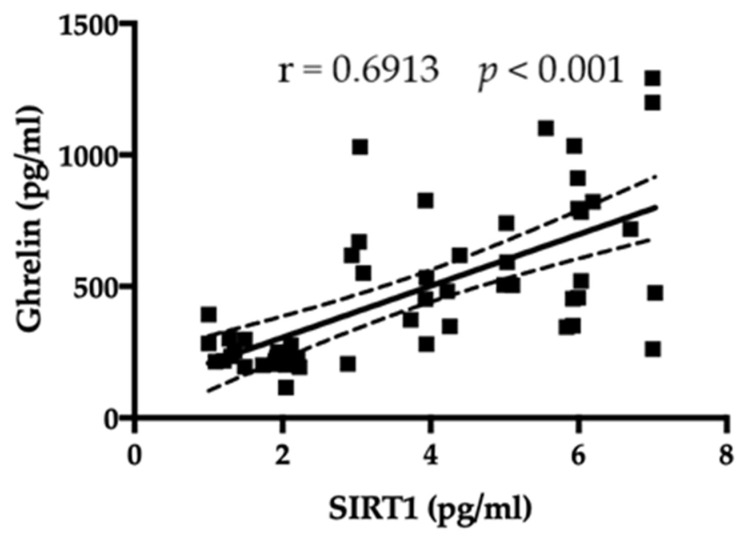
A positive correlation among amount of SIRT1 and ghrelin is evident.

On the contrary, a significant positive correlation was observed between serum levels of SIRT1 and ghrelin (r = 0.6913, *p* < 0.001; Figure 3).

**Figure 4:**
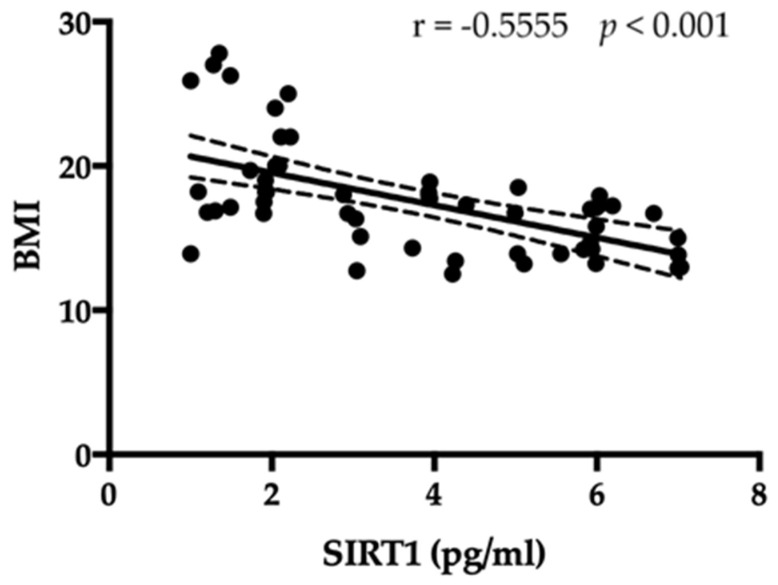
An inverse correlation among amount of SIRT1 and BMI is evident.

Serum amounts of SIRT1 were compared to the BMI of the patients with AN and controls. As shown in Figure 4, SIRT1 had a negative correlation with BMI (r = −0.5555, *p* < 0.001).

**Figure 5:**
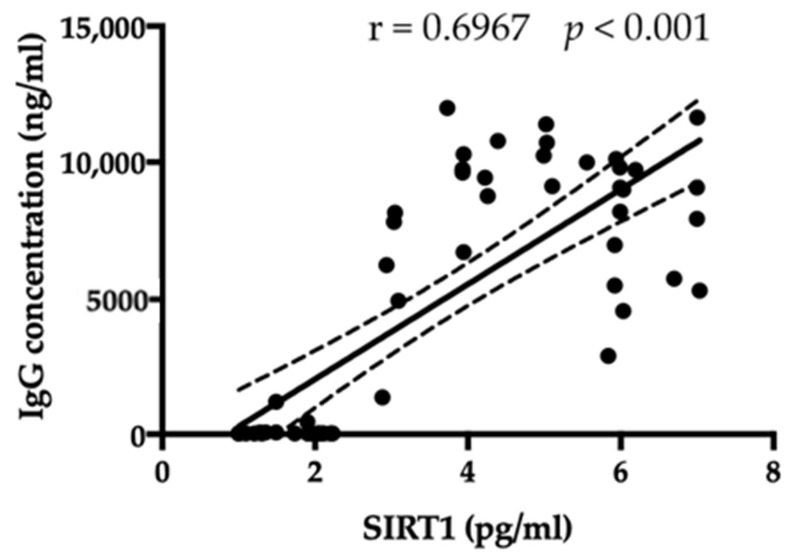
A positive correlation between SIRT1 and the amount of anti-hypothalamus antibodies exists.

Finally, a positive correlation between SIRT1 and the amount of anti-hypothalamus antibodies exists (r = 0.6967, *p* < 0.001, Figure 5).

## 4. Discussion

SIRT1 acts on the brain by regulating mood and behaviour. Human studies, though unbiased, seem to demonstrate the association of SIRT1 with anxiety states and several psychiatric disorders [37,46]. These data provide evidence of SIRT1′s key role in mood and behaviour modulation. SIRT1 is located in various different tissues and organs (i.e., liver, pancreas, heart, muscle, and adipose tissue) [47]. SIRT1 is also expressed in important metabolic centres of the brain, including ARC, ventromedial nucleus (VMN), dorsomedial nucleus (DMN) and hypothalamus paraventricular nucleus (PVN), the postrema area, and the nucleus of the solitary tract in the posterior brain [47]. Finally, SIRT1 is involved in the control of metabolic processes regulating body weight, such as the control of food intake, adiposity, and energy expenditure [47]. Early studies that analysed the role of SIRT1 in calorie restriction-induced longevity established SIRT1 as the molecular link mediating that phenomenon. Subsequently, several studies showed a higher content of SIRT1 in metabolically reactive hypothalamic nuclei in mice with reduced calorie intake [48]. Therefore, it can be inferred that the role of SIRT1 is to mediate responses to calorie restriction.

AN is a psychiatric disorder, and itse aetiology is not fully known. AN is characterised by lower than healthy body weight, distortion of body image, and fear of weight gain, often associated with persistent behaviours hindering weight gain [1,4,5,7]. AN is inserted as an eating disorder in DSM-5, and it has a significant risk of morbidity and mortality due to hemodynamic and electrolytic changes or suicidal behaviours [1,4,5,7,43]. The incidence of AN is increasing in developed countries, influencing particularly females, although recently the number of affected males is increasing, and its onset is often in adolescence or early adulthood [1,4,5,7]. The literature suggests that improving living conditions increases the risk of developing both autoimmune and allergic diseases [48]. Similarly, assuming autoimmune dysregulation, they could increase the risk of contracting AN [49].

In addition, it has been shown that caloric restriction induces hyperactivity in rodents [33] and that animals that overexpress SIRT1 are more active in stressful situations [34]. These observations are particularly interesting, since hyperactivity, or excessive exercise, is commonly observed in patients with AN. A recent study shows that after dieting, individuals predisposed to AN experience the perception of being much more active and alert [50]. This perception will result in an excited and high-mood state, which allows them to continue their daily life and consider their nutritional behaviour beneficial. However, patients get used to this state of hyperactivity, and they will have to starve progressively more, leading to the physical and cognitive deterioration [42]. The authors hypothesize that sustained and hyperactivated SIRT1 causes compulsive hyperactivity, the need for excessive exercise, and anxiety, which leads to further self-determination and increased activation of SIRT1. Interestingly, SIRT1 activity appears to decrease with age [51], making SIRT1 hyperactivation less likely in older people. This would be consistent with epidemiological data, showing the possibility of the development of AN with age [42]. In this context, our results seem to be aligned. Patients with AN were divided into two clusters, based on measured serum SIRT1 values, which appear to correspond to the duration of the disease: <3 years and >3 years. The average age of the most recent diagnoses is 20.94 years (range 18–28) and that of the most remote diagnoses 29.08 years (range 18–62). However, the analysis of the correlation between the amount of SIRT1 and the age of the patient did not show any kind of statistical significance. Finally, being related to physical activity, SIRT1 is involved in the development of addictions [35], anxiety [36,37], and depression [37,38]: all clinical aspects reported by patients affected by AN. All these elements reinforce the hypothesis that SIRT1 could play a key role in anorexic pathology. In addition, the upregulation of SIRT1 expression in anorexic rodent models [52] and an increase in SIRT1 levels in patients with AN has been recently demonstrated [41]. Leptin is an anorexigenic adipokine secreted by adipose tissue, working on the balance of body weight and in the psychophysiological processes associated with AN [10,53,54]. Leptin affects neuroendocrine and immune homeostasis, thermogenesis, reproduction, angiogenesis, and haematopoiesis, having a pleiotropic effect [53,54]. Serum leptin levels were decreased in patients with AN compared to healthy control subjects, returning to normal in the case of weight recovery [10,52,54].

Ghrelin is an acylated gastrointestinal hormone that is secreted mainly by the stomach in response to fasting [55]. Ghrelin is produced by enteroendocrine cells, and it is also known as the “hunger hormone” [55]. Ghrelin can stimulate nutritional and caloric intake, gastrointestinal motility, lipogenesis, blood glucose levels, lower blood pressure, and inhibit the release of the luteinizing hormone (LH) and follicle-stimulating hormone (FSH) [55]. Alterations in the secretion of ghrelin could have a fundamental role in the onset of AN [10].

Given these characteristics, the possible correlation between leptin, ghrelin, and serum expression of SIRT1 has been investigated.

In this study, the serum concentration of SIRT1 in relation to leptin, ghrelin, and BMI values in patients with AN and in healthy subjects has been evaluated. Our results show that SIRT1 blood concentration decreases progressively with increasing BMI, following a pattern consistent with that of leptin. The positive correlation of SIRT1 with ghrelin and the negative correlation with leptin was evident in both AN patients and the healthy controls. These findings suggest that peripheral evaluation of SIRT1 may be a possible clinical/biochemical parameter related to eating disorders.

Of interest, the duration of the psychiatric disorder could be related to a “return to normality” of these serum parameters. The improvement of the pathological state of patients (related to the duration of the illness) is accompanied by a rebalancing of SIRT1, leptin, and ghrelin, even if differences in the amount observed in sera among the two clusters of patients and, in comparison to healthy controls, are still not statistically significant (with the exception of SIRT1 amounts).

Another very fascinating point of discussion in research studies is the definition of the role of the immune system in the pathogenesis of AN. Recently, the presence of hypothalamus-reactive autoantibodies in the serum of patients affected by AN by a quantitative ELISA test has been demonstrated [10]. There is evidence of dysregulation of the immune system, such as bidirectionally linking autoimmune diseases with the onset of AN and vice versa [10,11]. However, to the best of our knowledge, no studies adequately demonstrate the common immunopathogenic mechanisms and relationship between autoimmunity and eating disorders, establishing the elements that could define the risk of a possible transition from one disease to another. In this study, a positive correlation between the amount of SIRT1 and the concentration of autoantibodies reactive to hypothalamic antigens has been observed. Of note, the duration of the disease also seems to correlate with a reduction in serum SIRT1 but not to IgG-specific values for hypothalamic antigens. These results suggest that SIRT1 has a relation to autoantibody production and may correlate with the intensity/severity of AN. Thus, reducing the production of specific autoantibodies for hypothalamic cells could be considered a possible sign of improvement in the clinical condition.

Additionally, in this case, the relationship with other autoimmune diseases is apparent: in rheumatoid arthritis, it has been demonstrated that there is a very significant positive correlation between SIRT1 and autoantibodies (such as the anti-cyclic citrulline polypeptide antibody and the anti-mutant citrulline vimentin antibody) [54].

The study should be read considering the following limitations: the relatively small sample size, especially regarding the few men enrolled, which does not allow the total generalized but are reported as preliminary findings. Secondly, for many years, eating disorders have only been studied in females, not in male patients. It is important to note that today, eating disorders (anorexia, bulimia, and especially binge-eating) are also increasing in the male population [55,56]. Considering the enrolment of different patients at different times, it was not possible to evaluate the different stages of AN in the same patient. However, to the best of our knowledge, this is the first study showing a variation of SIRT1, leptin, ghrelin, and IgG reactive to hypothalamic antigens in the clinical evolution of AN. Finally, several clinical and psychopathological conditions have not been investigated, such as mood state, hyperactivity, and presence of anxiety or physical activity, that could affect the immune system and SIRT1 of patients with AN.

## 5. Conclusions

Serum from patients with AN is characterized by a significant modification in the concentration of circulating SIRT1. Circulating SIRT1, which behaves consistently with leptin, ghrelin, BMI, and hypothalamus-reactive autoantibodies, and which changes during times, could play an interesting role for the clinical evolution of AN. It could be possible that interaction among SIRT1 and the other markers investigated plays a role in these pathophysiological conditions. Obviously, further studies testing whether SIRT1 could work as a potential target for the therapeutic approaches of metabolic disorders, or could help monitor the effect of novel medications to ameliorate the clinical conditions of patients affected by AN, are needed with longitudinal design.

## Figures and Tables

**Table 1:** Comparison between patients with AN and healthy control group in terms of sociodemographic and serum markers.

	Anorexia Nervosa (*N* = 32)	Control Group (*N* = 22)	*p*-Value
Gender (male/female)	2/30	4/18	-
Age (years)	24.77 ± 9.72	27.17 ± 8.61	0.448
Body Mass Index (kg/m^2^)	15.55 ± 1.98	20.57 ± 4.00	<0.001
**Serum markers**			
SIRT-1 (pg/mL)	5.18 ± 1.26	1.74 ± 0.42	<0.001
Leptin (pg/mL)	3715 ± 1994	13,831 ± 5917	<0.001
Ghrelin (pg/mL)	645.3 ± 271.9	234.5 ± 54.04	<0.001
IgG autoantibody to hypothalamic cells (ng/mL)	8476 ± 2267	167.5 ± 368.4	<0.001

SIRT1: Silent coupling information type regulation 2 homologous 1.

## Data Availability

Data that support the findings of this study and materials are available from the corresponding author, upon request.
